# An Effective Technique for Salvage of Cardiac-Related Devices

**Published:** 2012-01-24

**Authors:** Erin K. Knepp, Karan Chopra, Hamid R. Zahiri, Luther H. Holton III, Devinder P. Singh

**Affiliations:** Division of Plastic Surgery, University of Maryland Medical Center, Baltimore

## Abstract

Millions of patients require implantable cardiac devices for management of cardiac dysrhythmias. These devices are susceptible to erosion, exposure, or infection and plastic surgeons are consulted when salvage is required. As of yet, an anterior muscle-splitting approach to effectively and safely relocate the device into the subpectoral position has not been described in the plastic surgery literature. The authors retrospectively reviewed the charts of 7 patients who required repositioning of cardiac devices. Indications for repositioning included exposure, erosion, infection, hematoma at the time of primary placement (3), and one cosmetic revision. All patients were treated with subpectoral repositioning of the device into the subpectoral space via an anterior muscle-splitting approach. Six of 7 patients (86%) achieved successful long-term repositioning in the subpectoral position without recurrent exposure or hematoma and with good cosmetic results. One patient who had a prior history of multiple failed device placements required reoperation due to recurrent infection. The anterior muscle-splitting technique proposed by the authors for defibrillator or pacemaker salvage is a feasible technique with promising results. Plastic surgeons should be aware of this simple and effective approach.

The development of sophisticated medical technologies parallels the growth in American life expectancy. In particular, the evolving design of implantable cardiac devices such as pacemakers and automatic internal cardiac defibrillators has improved the management of patients prone to debilitating dysrhythmias. Every year, nearly 300 000 new patients in the United States require implantable cardiac devices.^1^ While these devices have undergone vast improvements in terms of design and size, they are foreign bodies nonetheless and thus susceptible to complications including exposure, pain, palpability, and infection. The reported incidence of infection ranges from 2 to nearly 20% of cases.^2^^-^8 These types of complications require either complete or partial removal and replantation of the device and often its associated leads.

Traditionally, cardiac electrophysiologists perform primary implantation of these devices. These practitioners tend to favor a subcutaneous site of implantation in the chest that obviates dissection of deeper tissues. While this approach is well tolerated by most patients and the subcutaneous space is conveniently accessible to the implanting electrophysiologist, it is not appropriate for all patients. For example, in patients who are cachectic, primary device placement into the subcutaneous space of the infraclavicular chest can lead to a visible deformity that may be unacceptable or intolerable (Fig 1).

In the case of device infection, pain, palpability, or exposure (Fig 2), plastic surgeons may be consulted for salvaging or reimplanting the device. Removal of the device can cause devastating complications such as venous, valvular, or atrioventricular (AV) injury, tamponade, and sudden cardiac death.^9^^,^^10^ The morbidity and mortality associated with device explantation may outweigh that of attempted salvage surgery.^11^

While other methods for in situ salvage exist, the authors implement a method of salvage that maintains the original access incision while safely placing the salvaged device and/or leads into a fresh and well-vascularized tissue plane. Specifically, this technique relocates the device beneath the pectoralis major muscle via an anterior muscle-splitting approach.

## METHODS

The authors retrospectively reviewed the charts of 7 patients who presented with impending or frank exposure, infection, cosmetic concerns, or hematoma at the time of primary placement by cardiology. All patients were treated with subpectoral repositioning via a muscle-splitting approach. The patients were treated over a 36-month period between August 2008 and August 2011 (mean follow-up, 21 months). The institutional review board approved this study.

## SURGICAL TECHNIQUE

**Figure 3:**
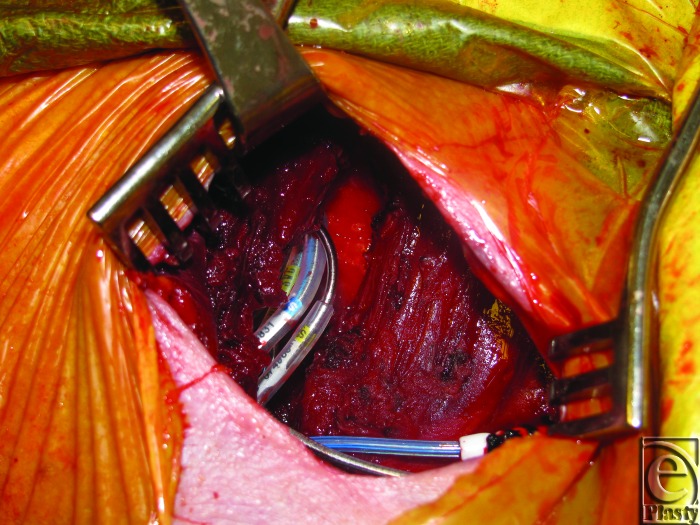
Anterior muscle-splitting approach to subpectoral place with generator replacement.

**Figure 4:**
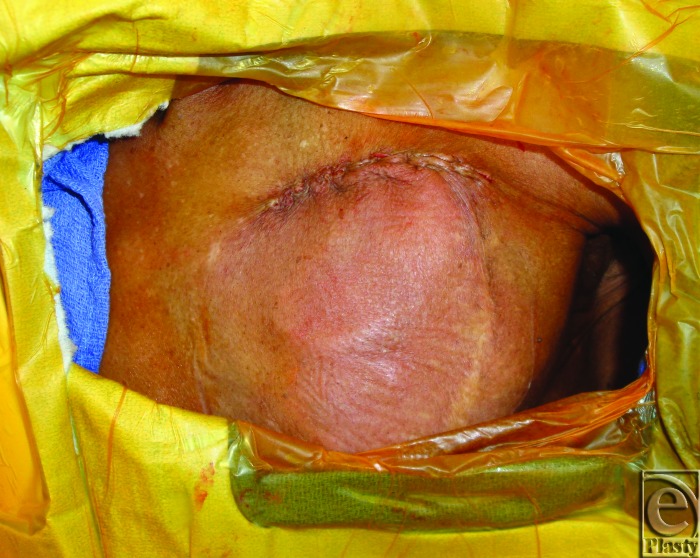
Immediate closure after subpectoral placement.

Patients underwent salvage surgeries under sterile technique with intravenous (IV) sedation and local anesthesia in the electrophysiology cardiology laboratory. Initial debridement of the pocket involved resection of necrotic skin and subcutaneous tissue overlying the old generator, and evacuation of any hematoma or periprosthetic infection, abscesses, or purulence. The surgical team then externalized and disconnected the devices from indwelling AV leads and performed a partial or total capsulectomy. The capsulectomy required meticulous dissection to skeletonize the leads, especially as they approach their entry into the subclavian system. After capsulectomy, the resulting pocket was copiously irrigated with antibiotic solution and inspected for hemostasis. Dissection was carried to the base of the pocket where the fascia of the pectoralis major muscle was encountered and the muscle was incised in the orientation of its fibers. A muscle-splitting approach was employed to divide the pectoralis major muscle and identify the subpectoral space. Once identified, a pocket was developed subpectorally using a combination of blunt dissection and electrocautery. At the base of the pocket, the pectoralis minor muscle was left unperturbed. For medial pockets or dissections, particular care was exercised to avoid disruption of neighboring perforators of the underlying internal mammary artery and vein. Throughout the process, awareness and preservation of the cephalic vein and identification of the pectoral branch of the thoracoacromial trunk are paramount. Once a satisfactory subpectoral pocket was created, a new cardiac electrical generator unit was placed and connected to the preexisting AV lead system (Fig 3). Next, the device was interrogated by the electrophysiology cardiology service. Muscle was then closed primarily over the device with 3-0 vicryl figure-of-8 absorbable sutures. A closed suction drainage catheter was left in the subcutaneous space and brought out through a remote incision. Meticulous layered closure of the subcutaneous tissue was performed to obliterate dead space in the original superficial pocket. Specifically, 3-0 vicryl absorbable sutures were to close Scarpa's fascia followed by interrupted 3-0 vicryl buried dermal sutures. Lastly, the skin was closed using a running 4-0 monocryl continuous subcuticular stitch (Fig 4). Incision was dressed using Steri strips, telfa pads, and finally tegaderm film.

## RESULTS

Between July 2008 and August 2011, a total of 7 patients were treated. There were 5 women and 2 men with a mean age of 59 years (range, 31-80 years old). In all cases, muscle-splitting technique was performed without intraoperative complications. The mean follow-up was 21 months (range, 9-35 months). Indications for subpectoral repositioning of the cardiac device included impending exposure (1), erosion (1), infection (1), hematoma at the time of initial placement by primary service (3), and cosmesis (1). Six patients (86%) achieved long-term successful repositioning of the generator in the subpectoral position with no recurrent infection or exposure, and with good cosmetic results. Only 1 patient had a failure of treatment due to a recurrent open wound and wire exposure 2 months postoperatively. In this case, failure was attributed to a 14-year-old lead that was not exchanged at the time of device salvage. This patient had a history of several failed device placement in bilateral subcutaneous locations secondary to infection requiring replantation by the cardiac electrophysiology service. After the authors salvaged the device with subpectoral repositioning, recurrent infection developed with a positive wound culture for *Enterobacter cloacae*. Because of the recurrent history of infection with device placement in the chest wall, the authors chose to place a new device and lead system in the abdominal subcutaneous tissue of the right lower quadrant. The patient was successfully treated with IV antibiotics and did not develop subsequent infection.

## DISCUSSION

Subpectoral positioning of cardiac devices is not a novel concept. In fact, techniques for subpectoral positioning of cardiac devices using a *lateral* approach have been described.^12^ Furthermore, comparison of primary placement of cardiac devices in the subpectoral space versus the traditional subcutaneous space was investigated.^13^ Results demonstrated no significant differences in freedom from complications. Thus, the current practice adopted by most electrophysiology laboratories is to place these devices into the subcutaneous space allowing shorter procedural time and requiring less demanding technical maneuvers. Cardiac electrophysiologists can easily and quickly access the subcutaneous space without the need for plastic surgery consultation.

There is a paucity of literature on the management of infected devices or devices that are exposed because of either erosion through the overlying skin or secondary to poor wound healing. Device explantation is often not feasible because of risks including sudden cardiac death. For this reason, salvage by repositioning of these infected or exposed cardiac devices often becomes imperative in managing patients with debilitating cardiac arrhythmias.

Less data has been published on techniques used for device salvage in the setting of infection or device erosion. Several authors have independently reported successful outcomes with treated infected device pockets. Taylor and colleagues^14^ reported the treatment of pacer pocket infections with revision and placement of a continuous irrigation system. Hurst et al treated infected device pockets in a similar fashion with the use of closed antibiotic irrigation for lead preservation. Lee's group reported success with treating infected device pockets via revision followed by placement of continuous irrigation system and closed antibiotic irrigation.^15^^-^17 These approaches, however, do not move the device to a sterile plane of tissue; thus, long-term sterility after discontinuation of the antibiotic irrigation systems is questionable.

Another group, Kolker et al,^18^ presented 6 patients treated with debridement, capsulectomy, and local rhomboid skin flap closures; 83% achieved long-term successful salvage. This technique carries the risk of additional donor site morbidity since adjacent tissue must be harvested and rotated into the primary defect. Yamada reported successful salvage of AV lead systems, in 17 of 18 patients, using pocket debridement, iodine packing, and creation of an entirely new remote pocket.^19^ A success rate of 74% with pocket debridement, lead preservation, and creation of a new ipsilateral subcutaneous pocket in patients with negative wound cultures was noted by Griffith.^20^ With these strategies, however, the risk of infection, erosion, or exposure may not be significantly reduced as the cardiac device is still placed within the prepectoral space. Har-Shai et al^21^ described a technique for subcapsular relocation of the generator and lead systems. While this may be an effective salvage method, subcapsular tissue coverage is less bulky and durable than the pectoralis major muscle, making this technique subject to more complications.

As mentioned previously, subpectoral positioning for device salvage has been described. In 1995, Foster^12^ described successful subpectoral placement in 6 patients, using a lateral axillary approach. Soon after, Jenson^22^ described a case report of the repositioning of an implantable generator from an abdominal pocket to a subpectoral location, using an axillary tunneling technique. In 2004, Kistler et al^23^ reported on anatomic findings discovered upon device recall surgery, implying that implant location was, in fact, “intrapectoral,” located in a medial position between the 2 heads of pectoralis major muscle.

Most recently in 2010, Al-Bataineh et al^24^ described a lateral axillary approach to the subpectoral plane in patients with ipsilateral prepectoral infection and limited venous access. They reported no recurrence of infection, but one hematoma and one pneumothorax in 16 patients treated. Most importantly, techniques describing a lateral axillary approach have lacked a discussion regarding the effect on nipple sensation, which is a concern in any lateral axillary pectoral approach as the anterolateral cutaneous branch of T4 intercostal nerve is at risk here.^25^^,^^26^

Our series demonstrates comparable overall success with true subpectoral positioning of implantable cardiac devices. Analysis of our 7 cases, with indications related to impending or frank exposure, infection, hematoma, and cosmesis, revealed an 86% success rate with subpectoral repositioning with 21-month mean follow-up.

Important advantages of the anterior muscle-splitting technique include the ability to reposition the device into a clean plane, coverage with healthy vascularized muscle, and that even with debridement of necrotic skin and subcutaneous tissue, skin of the original pocket is easily closable over drains by means of simple undermining without the need for local flaps. The results demonstrated no hematoma or pneumothoraces, and nipple sensation is not at risk with this approach. All surgeries were performed safely under IV sedation with local anesthesia in the electrophysiology laboratory allowing for easy access to fluoroscopy and interrogation software. This facilitated a collaborative approach between cardiology and plastic surgery allowing for device exchange and subpectoral placement simultaneously in the electrophysiology laboratory.

Like previous reports of secondary repositioning of cardiac devices into the subpectoral position, our series is retrospective and our sample size is low. Therefore, it is difficult to draw stringent conclusions. Our results match existing data outcomes and demonstrate a safe approach to the subpectoral space with minimal morbidity.

As in the treatment of any infected prosthesis, if explantation of an infected device is not possible, then aggressive debridement, obliteration of all dead space, and coverage with healthy vascularized tissue such as muscle flaps are essential principles of management. There is a wide spectrum of treatment options (see Table 1), and reconstructive choices should be tailored to individual clinical situations. Our series demonstrates that salvage of cardiac devices with anterior muscle-splitting subpectoral repositioning is a technically feasible approach with favorable outcomes and low morbidity. Plastic surgeons should be aware of this simple and effective approach.

## Figures and Tables

**Figure 1:**
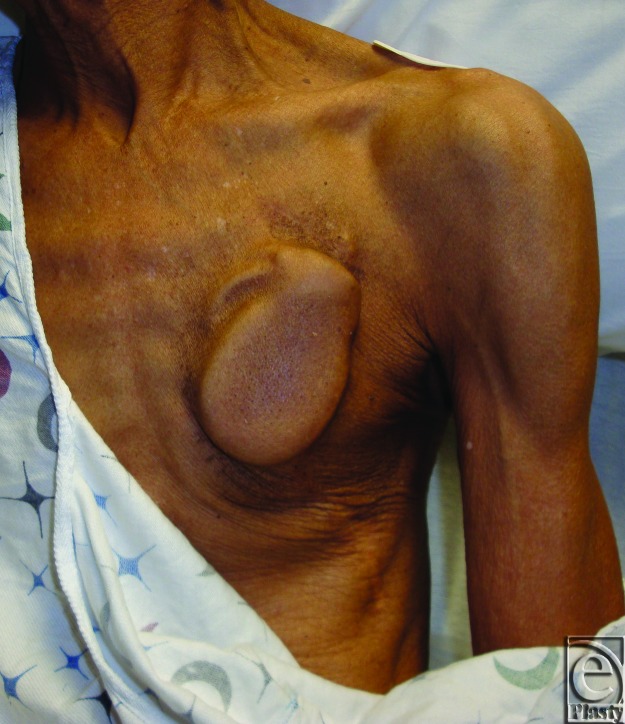
Impending generator exposure in cardiac cachexia.

**Figure 2:**
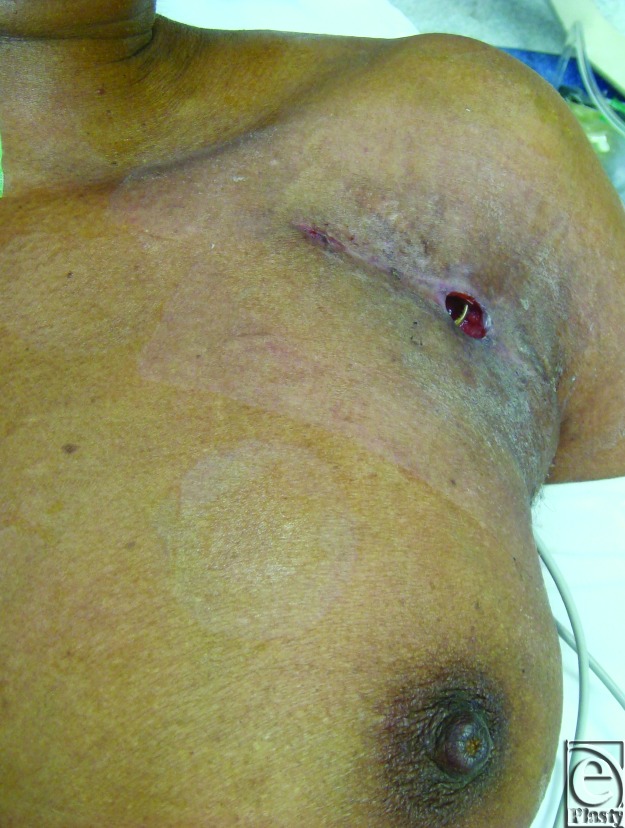
Exposure of atrioventricular lead.

**Table 1:** Summary of management options

Device explantation and externalization
Debridement and local wound care with closed irrigation system
Revision of subcutaneous pocket and local skin flap closure
Repositioning to distant subcutaneous location
Repositioning to subcapsular location
Repositioning to intrapectoral location
Repositioning to subpectoral position by axillary approach
Repositioning to subpectoral position by anterior muscle-splitting approach
